# A Case of Pulmonary Tuerculosis Treated by Artificial Phemothorax

**Published:** 1910-11

**Authors:** Mary E. Lapham

**Affiliations:** Highland Camp Sanatorium, Highlands, N. C.


### A CASE OF PULMONARY TUBERCULOSIS TREATED BY ARTIFICIAL PHEMOTHORAX.

By Mary E. Lapham, M. D.
Highlands Camp Sanatorium, Highlands, N. C.

*Concluded.*

The patient gained twenty pounds in weight, her appetite returned, she felt well and strong, and improved steadily until the time for menstruation in January, 1910, came. At this time there were no physical signs below the third interspace in front, and the fourth in the back. Menstuation did not occur in January. The cough became distressing, temperature rose to 100, and rales appeared down to the sixth interspace, accompanied by harsh inspiration and expiration. In March the lung had pretty well dried out again to the third and fourth spaces. On the tenth of April she began spitting blood, on the twelfth of April there was a profuse hemorrhage, followed by a temperature of 104, paroxysmal coughing, vomiting, inability to retain food, etc., etc., and menstruation was again established. The extension of the pneumonic process was so marked in the lung, and so indisputably associated with pelvic influences that it was decided to protect the lung from future hemorrhages and further advances of the process by compressing it with nitrogen injected into the plueral cavity.

Turban says (1) "In amenorrhoea as well as in delayed menstruation we often see at the time when menstruation should occur, very troublesome congestions of the lungs, and even fatal pneumonic extensions of the process. These cases are especially associated with toxic amenorrhoea, and the hemorrhages occurring at this time have been regarded as vicarious menstruation since earliest antiquity. We have a genuine circulus vitiosus. The disease interferes with menstruation, and the efforts of the organism to bring about menstruation in spite of opposing conditions have a very injurious effect upon the disease."

"The influence of menstruation upon pulmonary tuberculosis is indisputable. Fonsagrives (2) compares the vascular network of the lungs and of the uterus to the two globes of an hour

NOTE: Owing to an oversight of the Printer, this Report of Cases was omitted from the October issue of the Journal.

glass. In many cases the effect of menstruation is not marked, and not especially injurious, but in many others the whole course of the disease is unfavorably dominated. The powerful influence of the vaso-motor depression associated with menstruation is most felt in the affected regions, in the centers of least resistance. On the other hand, tubercular toxins are powerful vaso-motor depressants, and so we have a summation of two injurious influences. Menstruation causes a fall in blood pressure, a lessening of resistance throughout the female organism, due to vaso-motor relaxation. This leads to congestion in areas where the blood vessels are damaged and unable to withstand the fall in blood pressure, so that stasis results. These congestions may be seen in any tissues affected by tubercular toxins, in the mucous membranes of the throat, the larynx and trachea. The patients say they have "caught cold," they complain of breast pains, and oppression. The cough and expectoration are increased. There may be coryza, acute pharyngitis, laryngitis, trachitis, bronchitis, even dry pluerisy. The negative sputum becomes positive, and there is a rise in temperature. Physical examination shows that the areas of dullness are more intense, impure breathing gets rougher, suppressed breathing may become bronchial, the rales are increased and more resonant. In cases of latent tuberculosis of the lungs the effects of menstruation may be compared to a tuberculin reaction. During menstruation, or closely associated with it, there may be bronchopneumonia and extensions of the tubercular processes in the lungs, which are of serious prognostic import. They may persist as Sabourin (3) has reported, for fourteen days, thus allowing only fourteen days for recovery, and rendering an arrest of the process almost impossible."

On the 24th. of April, 1910, in the Mercy Hospital in Chicago, Dr. John B. Murphy, author of the method of artificial pneumothorax in America, (4) introduced 60 cu. in., of nitrogen into the left pleural cavity. The patient lay on her right side, with the left arm thrown up well over the head. The skin was painted with tincture of iodine over the fourth axillary interspace, anesthetized with ethyl chloride, and a slight incision made through the skin, large enough to admit a medium sized aspiration needle, which was thrust through the muscles and pleura. After puncturing the pleura no air could be heard entering the head of the needle, and it was inferred that the pleural surfaces were adherent. A second attempt also failed, but the third was successful, and 60 cu. in., of nitrogen were induced to enter, mainly by the inspiratory efforts of the patient, as there was no vis a tergo to force the nitrogen out of its container.

The temperature rose to 101.4, pulse went up to 130, the cough was almost persistent, and the amount of expectoration markedly increased. These effects gradually subsided, and on the 28th. of April 55 cu. in. of nitrogen were introduced. On May 1 the temperature was normal, patient ate and slept well, coughed and raised very little. On May 3 57 cu. in. of nitrogen were admitted, and on the 4th of May the patient returned to the Highlands Sanatorium, where the injections were continued according to Forlanini, in small quantities—300 to 400 cc, and often—one o twrice a week. At the present writing (June 12) the temperature is normal, pulse 96, the bacili and alveolar cells have entirely disappeared from the sputum, the patient is up and about, eating well, sleeping well, raising almost nothing. The X-Ray shows the lung well compressed up against the hilum, and it is hoped that a conversion of the tubercular lesions into cicaltricial tissue will bind the lung so firmly together into an organized mass that no infections process can survive therein. The great results of compression of the lung by nitrogen injected into the pleural cavity are first, the forcing of all extraneous material out through the bronchial tubes, and the mouth; second, the effects of the pressure on the collapsed lung tissues forms an extraordinary overgrowth of connective tissue, which converts the diseased lung into a cicatrized mass. When this anatomical recovery is established there can be no further danger of a relapse, and there can be no further extensions of a tubercular process, for they are all crushed out.

Forlanini (5) reports authorsies of cases treated by artificial pneumothorax, and dead of other diseases, in which the compressed lung was found to be a hard, dry mass of connective tissue, absolutely devoid of any trace of tubercular processes, and the anatomical recovery in these cases is a permanent one.

1 Turban. Menstruation and lungentuberkulose, Davos. 1909

2 Fonsagriues. Therapelutigue de la Phthisie pulmonaire, 2 Edition, Paris 1880.

3 Sabourin. La fieure menstruelle des phdhisiyures. Revure de med. Mars. 1905.

4 Murphy. John B. Surgery of the Lung. J. A. M. A. 1898.

5 Forlanini. Cura della Tisi Polmonare col Pneumotorace prodotto artificialmente, Pavia, 1908.

